# What are the experiences of medical students and their trainers regarding undergraduate training in primary health care at four South African medical schools? A qualitative study

**DOI:** 10.3389/fmed.2024.1337140

**Published:** 2024-06-18

**Authors:** Langalibalele Honey Mabuza, Mosa Moshabela

**Affiliations:** ^1^School of Medicine, Clinical Integrated Programs, Sefako Makgatho Health Sciences University, Pretoria, South Africa; ^2^Research and Innovation Department, University of KwaZulu-Natal, Durban, South Africa

**Keywords:** primary health care, training experiences, UG medical students, student trainers, generalists, specialists

## Abstract

**Background:**

In 1978, the World Health Organization (WHO) adopted primary health care (PHC) as the most effective strategy to meet the healthcare needs of communities. This raises the question as to the extent and nature of the training that undergraduate (UG) medical students receive in medical schools regarding PHC, following this statement.

**Aim:**

The study aim was to explore the experiences of UG medical students and their trainers regarding training in PHC in their institutions.

**Methods:**

A qualitative study was conducted among UG medical students (MBChB 4-6) and their trainers at four conveniently selected South African medical schools. A total of 16 focus group discussions (FGDs) and 27 in-depth interviews were conducted among students and their trainers, respectively. The MAXQDA 2020 (Analytics Pro) software program was used to arrange the data, resulting in 2,179 data segments, from which categories, sub-themes and themes were derived.

**Results:**

Both the UG medical students and their trainers regarded PHC as mainly an approach to health rather than a level of care. Students were trained by specialists and generalists, received training in the undifferentiated patient, coordinated, comprehensive and continuity of care. The training in tertiary centers, conducted mainly by specialists, the implicitness of the training and the inadequacy of trainers at the PHC settings presented challenges.

**Conclusion:**

Students and their trainers experienced UG student training in PHC in line with the internationally recognized principles on the subject. The view by students and their trainers that PHC is an approach rather than a level of care enhanced its training across disciplines. The implicitness of the training and the tertiary learning platforms were the main challenges experienced. For optimum PHC training, more time should be dedicated to distributed training platforms with supportive specialist outreach programs in the South African medical schools.

## Background

In 1978, the World Health Organisation (WHO) adopted primary health care (PHC) as the most effective strategy to meet the health care needs of communities (1). Furthermore, in 2018 the WHO advanced a vision for PHC in the 21st century, which states that “Primary Health Care is the whole-of-society approach in health, aimed at providing equitable health and well-being to individuals, families and communities, as early as possible in the continuum of health, namely health promotion, disease prevention, curative, rehabilitative and palliative care, as close as feasible to people’s day-to-day environments” (2). Literature indicates that PHC is generally described in terms similar to the WHO definition of PHC, as “taking the whole society into consideration” (3, 4); “providing health and well-being” (3); “addressing the whole spectrum of ailments” (3); and “accessibility to communities” (3, 5).

Since the dawn of democracy in South Africa in 1994, the government has achieved several successes in the implementation of PHC. The 14 health administrations of the Bantustans were consolidated into one national department comprising nine provincial health departments. Health facilities were de-segregated and PHC, delivered via a district health system, has been made the cornerstone of the country’s health policy (6). Furthermore, the South African government introduced the PHC re-engineering plan in 2010, a three-stream approach to PHC comprising: ward-based PHC outreach teams, strengthening school health services and district-based clinical specialist teams with a focus on improving maternal and child health (7). Logically, the implementation of the PHC re-engineering plan would require its incorporation in the training of the South African medical students. There is already an explicit social accountability imperative for medical schools in the country to produce undergraduates who are equipped to function effectively at PHC, regardless of who trains them (8, 9). A conference statement released by the Eighth Primary Care and Family Medicine Education (PRIMAFAMED) conference held in Nairobi in 2016, attended by 18 African universities, acknowledged that PHC training in the African region is only centered at post-graduate level whereas it “should begin during undergraduate training” (10).

Barbara Starfield proposed four pillars of PHC, namely first contact care, continuity of care, comprehensiveness, and coordination of care (5). Emanating from these pillars, the principles of PHC are generally understood as (a) the main entry point and interface of the population and the health system (11), whereby PHC plays the gatekeeping and access functions; (b) a broad-based healthcare incorporating health promotion, disease prevention, curative, rehabilitative and palliative care throughout the life of a patient (2); and (c) interprofessional collaborative patient care provided at all levels of care addressing patients’ social determinants of health (including their social, economic, environmental and educational situations) (12); and (d) building relationships with the patient for patient-centered care (13). In the current study, the researchers factored in the four pillars of PHC in their exploration of the experiences of students and their trainers in PHC training, and the challenges encountered in the process.

The current study was nested in the multicenter collaborative study named “Transformation in Medical Education” (TiME), in which four universities collaborated: University of KwaZulu-Natal (UKZN), Sefako Makgatho Health Sciences university (SMU), University of the Witwatersrand (WITS) and Walter Sisulu University (WSU). The objective of this umbrella study was to investigate the balance between specialist and generalist training platforms with particular emphasis on medical students’ training in the South African medical schools (14).

Literature indicates the importance of training medical students in PHC, equipping them with knowledge and skills for the field of practice (15). At the time when the current study was conducted, the main focus in South Africa had been on exposure of UG medical students to PHC through distributed training platforms (16). Recently, literature exploring the understanding of students and their trainers regarding PHC has been published (17). However, the experiences of students and their trainers in PHC training has not been explored. Since the training of students in PHC is conducted by both generalists and specialists in South African medical schools, there is need for both to work collaborately and assume a common approach for the task. In answering the research question of the current study which is on the “experiences in PHC training,” the position of the specialists and generalists may be ascertained, opening a window of opportunity to address shortfall and discrepancies among them. The findings of this study could add another dimension to the body of knowledge on the subject.

## Materials and methods

### Study context

This paper is part of the principal researcher’s PhD project which explores the training of UG medical students in “generalist medical practice” and “primary health care.” He has produced four manuscripts each addressing a specific objective of the project focusing on the medical students and their trainers’ (a) understanding of “generalist medical practice,” (b) experiences in the training in “generalist medical practice,” (c) understanding of “primary health care” and, lastly (d) experiences of the training in “primary health care.” The current study addresses objective (d).

### Researcher’s positionality

As a family physician at the time of the study, the principal researcher was a trainer of UG and postgraduate students in the discipline of Family Medicine. He was based at SMU, one of the four settings where the study was conducted. It became important for him to declare this position to both students and fellow trainers at the beginning of each interview. This was followed by the explanation of the study, including its aim and objectives. Specifically for students, they were requested to respond with honesty as there were no right or wrong responses. They were also informed that they were free to express their views, even those they would have thought would go against the interviewer. They were assured that no response would be found unacceptable to the interviewer. Furthermore, the interviewer adopted a reflexive approach (maintaining self-consciousness) during data collection, analysis and interpretation—as recommended in literature (18).

### Study design

A qualitative phenomenological design as described in literature (19) was used to explore the experiences of UG medical students and their trainers in PHC training. This design was regarded as appropriate for this study since a phenomenological enquiry elicits the lived experiences of individuals in a particular group (20).

### Study setting

At the time of the study, there were nine medical schools in South Africa. All were invited by UKZN to participate in the TiME study (14) and funded by the National Research Foundation of South Africa (NRF). However, only three accepted the invitation: WSU, SMU, and WITS.

### Data collection

Data collection was conducted by the principal researcher with the assistance of a final year medical student who had been trained in research methodology (including data collection) by the first author and his PhD supervisor. Data collection from the students’ trainers was conducted through in-depth, one-on-one interviews, while focus group discussions (FGDs) were use for the students. Heads of departments or their course coordinators in the various disciplines were purposively recruited to participate. During the interviews, the opening statement for each trainer was: “As a trainer of undergraduate medical students, what has been your experiences in training them on primary health care?” The prompts have been listed in Table 1. Regarding students, the exploratory statement used for each FGD was: “As undergraduate medical students in this institution, what have been your experiences regarding your training in “primary health care?” The prompts have also been listed in the table referred to above. The prompt for both students’ trainers and the students were derived from the study objectives. Each in-depth interview and FGD lasted for about 45 min on average. Twenty-seven students’ trainers and 16 FGDs (comprising 102 students) participated in the study. Data collection was conducted over a four-year period (2016 to 2020).

**Table 1:** The interview guide used in data collection.

	Section A: Students (FGDs)	Section B: Trainers (in-depth interviews)
Exploratory question	“As undergraduate medical students in this institution, what have been your experiences regarding your training in “primary health care”?	“As a trainer of undergraduate medical students, what has been your experiences in training them on “primary health care”?
Prompts	Tell us about your experiences of PHC regarding the following: Your trainers in PHC Primary health care as: As an approach and/or level of care The first point of patient contact Comprehensive care Coordination care Continuity of care	Tell us about your experiences in training students on PHC regarding the following: Your involvement as the trainer of PHC Primary health care as: An approach and/or level of care The first point of patient contact Comprehensive care Coordination care Continuity of care
	As students, did you encounter challenges in your training on PHC? Tell us more.	As the trainer, did you encounter challenges in training the students on PHC? Tell us more.

The students were from the fourth (MBChB 4) to the final year (MBChB 6) whom the researchers regarded appropriate as they were in their clinical years of training and most likely to have experienced clinical training in PHC. Each FGD comprised five to eight students. In each medical school, four FGDs were arranged: three homogeneous (comprising students in the same year of study from 4th to 6th year), plus one heterogeneous group (comprising mixed year levels from the 4th to the 6th year). The heterogenous group, the FGD consisted of about two students per year group. The heterogenous group was arranged to capture the interaction among students from various year levels in a single group.

### Data analysis

The principal researcher used both the inductive and deductive methods of data analysis (21). As indicated in the interview guide (Table 1), there was an unstructured exploratory question for both the one-on-one and FGD interviews which gathered broad information on the experiences in PHC training, for which the inductive analysis method was used (22). This was followed by the open-ended prompts as a follow-up enquiry on matters which a participant had not initially addressed when responding to the broad exploratory question. For the responses to the semi-structured prompts, the deductive method was used (23). The last prompt, which enquired on “challenges experienced” in PHC training was analyzed through the inductive method (21). The analysis was thematic guided by the open-ended questions in the interview guide. The questions were based on Barbara Starfield’s proposed four pillars of PHC, namely comprehensiveness, first contact access, coordination and continuity of care (24). All interviews were digitally recorded and transcribed verbatim by a linguistic expert transcriber. Each transcript formed the basis of the data analysis. The authors used the MAXQDA 2020 (Analytics Pro) software program to arrange the data which yielded 2,179 data segments.

### Theoretical framework

Vygotsky’s Social Constructivist theoretical framework (SCT) (25) as well as Lave and Wegner’s Situated Learning theory (SLT) (26) were used as the lens through which this study was conducted. Both theories contend that in the learning setting, there are the “more knowledgeable others” (experts) who train the “novices” (apprentices) to become experts in “the community of practice,” and that learning occurs best within the context of its application (27).

Both theories assume a constructivist approach whereby the learner is given knowledge and skills enabling him/her to solve problems independently. Prior to implementation of the skills and knowledge for independent implementation of PHC principles, the students first needed to gain understanding of the concept, as imparted to them by their preceptors. The researchers in this study, in turn, needed to explore the experiences of students and their trainers in PHC training, hence the choice of the phenomenological study design in relation to the theoretical frameworks. In a similar manner, it became appropriate to conduct data collection by means of interviews: FGDs and in-depth interviews for the students and their preceptors, respectively as these also followed the exploratory trend.

For data interpretation, the lens provided by these theoretical frameworks assisted the researchers to compare the information given by both groups and discuss the findings looking at “both sides of the coin.” For example, what information did each group provide regarding PHC as “the point of first contact for patients”? Furthermore, it was observed that the students’ trainers related with their students as the “more knowledgeable others” moving the former from the position of “novices” to the position of “experts” (25, 26). Thus, throughout the study, the theories gave insight to the researchers in exploring the experiences of the students and their trainers regarding PHC training.

### Trustworthiness

In this study, trustworthiness was ensured by consideration of credibility, dependability, confirmability, and transferability as explained in literature (28). Credibility was ensured by the creation of a dataset (see Supplementary materials), containing the study findings for member checking regarding the accuracy of the information captured. Data reproducibility (dependability) was ensured by providing a thick description of the study methods (29).

Confirmability, pertaining to objectivity of the researcher in collecting and interpreting data (30), was ensured by reflexivity. During interaction with the participants, the researcher was self-conscious of his influence on them as a trainer of medical students himself. He allowed independent expression among all participants. Transferability, the degree to which the study findings are applicable to similar settings (28), was ensured by providing thick description of study methods, setting and description of the participants (22). Data triangulation was achieved through field notes taken during the interviews and reference to the students’ training manuals obtained from each medical school (30).

## Findings

We identified seven major themes on the experiences on PHC training by UG medical students and their trainers (Table 2).

**Table 2:** Themes and sub-themes on the experiences of medical students and their trainers in PHC.

A. Students		B. Trainers	
1.	PHC—an approach to patient care	1.	PHC—an approach to patient care
2.	Training by generalists and specialists	2.	Training by generalists and specialists
2.1.	Generalists	2.1.	Generalists
2.2.	Specialists	2.2.	Specialists
3.	First contact	3.	First contact
4.	Comprehensive care	4.	Comprehensive care
5.	Coordination of care	5.	Coordination of care
6.	Continuity of care	6.	Continuity of care6.
7.	Challenges experienced	7.	Challenges experienced
7.1.	Training in tertiary health facilities	7.1.	Training in tertiary institutions
7.2.	Practical application of PHC lacking	7.2.	Lack of trainers at distributed platforms
7.3.	Training in PHC implicit		

### Students

Students described their experiences in the training in PHC by identifying their trainers, PHC as the first contact of patient care, comprehensive care, coordination of care and continuity of care. They also narrated the challenges they had experienced during the training.

#### Experience of PHC as an approach

Students indicated that they had experienced PHC as an approach rather than a level of patient care, and as an approach, they had observed that some specialists (not only the generalists) had practiced PHC in their various disciplines by educating patients on disease prevention, which indicated to the students that PHC cuts across all disciplines and is not confined to the patient entry levels which were the clinics and other community based health facilities.

*Yeah, with the primary health care being a care, not a level, … most specialists do practice it, I have experienced [it] in the surgery block where they educated the patient about the, the prevention of a condition. Also, in psychiatry also I had experienced it,…* (SMSM4.1, MBChB 4, female student, 21 years).

#### Generalist and specialist trainers in PHC

In their experiences of being trained by generalists and specialists, students compared these experiences.

##### Generalists

According to the students, generalist practice trainers offered training more than specialists in PHC. However, they also felt that PHC should be trained by all disciplines and not only confined to generalist trainers. This displayed their understanding that, although generalist trainers were advantaged by their training environment (PHC settings), the principles of PHC were also applicable in specialist disciplines.

*…it's essentially the general practitioner who has a greater caliber of skills to be able to address primary health care as opposed to the surgeon.* (WTSM5.2, MBChB 5, male student, 22 years).

The discipline of Family Medicine was identified as particularly giving students the opportunity of exposure to PHC.

*I think it’s mostly Family Medicine, honestly speaking, our Family Medicine rotation is the one that focusses a lot on it [training in PHC]. In fourth year, we are in clinics, in community health care centres and then in fifth year we are in a district hospital but in a rural area.* (KZS5.1, MBChB 5, male students, 23 years).

However, students expressed further opinion on the responsible disciplines in training students on PHC.

*It [teaching primary health care] is supposed to be a sort of shared responsibility among the departments.* (SMS5.4, MBChB 5, female student, 23 years).

##### Specialists

Some students had experienced being trained by specialists on PHC, through reconstruction of the state of the patient on arrival at PHC before referral to specialized disciplines. However, some students also indicated that they had not experienced training in PHC from specialist disciplines because of the specialized setting. Students had the ability compare and contrast the training on PHC they had received from specialists and generalists and managed to state their experiential differences.

*In my case when we went there, they did [train us on PHC]. They often asked us "what would lead to this? And what did you expect to see before the patient came in?". And then there are certain signs that started presenting before, which could give you a clue towards the diagnosis.* (WTS4.3, MBBCh 4, male student, 24 years).

*So, they often asked us to, to not only think about what the patient is presenting with now, but also what they might have presented with before, so that … in case we saw a patient at a primary health care setting, that we would uhm… have enough knowledge to pick up a case and either treat it or refer it upwards.* (WTS4.4, MBBCh 4, female student, 21 years).

*because obviously if you're doing an Obs and Gyne [Obstetrics & Gynaecology] block for example, and you’re in a Gyne [Gynaecological] ward, … you don't really want to explore the other problems.* (KZS4.5, MBChB 4, female student, 26 years).

#### First contact

Students pointed out that they were given guidance on the approach to patients seen at PHC settings at entry points into the healthcare system. They were taught to apply basic principles of recognition of a condition, stabilization of emergencies and appropriate referral determined by limitations. It became clear to the researchers that the students had understood the scope of practice of a generalist medical practitioner in terms of skills and available resources at PHC settings, beyond which they were to refer the patient to more specialized centers.

*[At primary health care] you have to be able to recognize these conditions and refer where possible. Yeah, and up to so far, yeah, they emphasize on that, yeah, we are being taught that when it gets here, there's nothing you can do … make sure that they’re breathing, give them oxygen, give them fluids and then refer, …* (SMS5.5, MBChB 5 female student, 29 years old).

#### Comprehensive care

Training in comprehensive care entailed consideration of other factors that could influence the patient’s wellbeing: the social determinants of health. This enabled the students to address the patient’s condition comprehensively, not to lose focus on all the aspects that could affect their wellbeing. Students had developed the sense of appreciation that a patient should be treated contextually and not as a single individual, because unwellness goes beyond the clinical condition.

*Yeah, we do get trained on comprehensive patient care. It's about treating the patient, everything that has to do with the patient. So, it's not only treating the disease – the biological part of it. So, you consider the social background of the patient, you consider the psychological issues that they might have also. So, if the patient, say you are treating an HIV or TB patient, … maybe they live in a house where they can infect other people, where they can infect children. So, you need to also deal with that, the social background.* (WTS5.3, MBBCh 5, female student, 25 years).

*So, for social determinants of health, we get taught to include a social history, and a social history will include things like … family history. But we also need to make sure that we look at the patient holistically in terms of occupation, finances, access to clean water, electricity, and all of those things that can affect the patient’s health in the long term.* (SMS6.3, MBCHB 6, male students, 28 years).

#### Coordination of care

Various disciplines trained students on collaborative patient care, giving them insight on which patient’s condition should be attended to by which discipline, including receiving back patients who had initially been referred to other disciplines. This aspect of students’ experience of PHC training gave the researchers an indication that the students had gained insight into the teamwork in patient care, which could only function well under proper coordination.

*…they do teach us … to be able to identify different conditions, … knowing who is responsible or who can help on which particular condition and where is that person. So, you need to know and understand what a dietitian does, you need to know and understand what all these specialists to do. We know if I have a patient who is a diabetic … they need to change their lifestyles, I have to refer them to someone who will give them proper exercises and [diet] schedules …* (WTS5.5, MBBCh 5, male student, 23 years).

*We are also taught that you don't only send them, you also have to check on their progress while they are still there [where you referred them to] because they'll have to come back to you again after they are done.* (KZSM5.2, MBChB 5, male student, 23 years).

#### Continuity of care

Continuity of patient care entailed arrangement of patient follow-up. GP attachment gave them the opportunity for training in continuity of care. The students had developed the sense of a life-long relationship between a patient and her/his clinician. The researchers could appreciate that students understood the meaning of being a clinician.

*Well of course we are trained to … make follow-up, so we ask them [patients] to come back after [a] certain period of time based on the condition … they are having problems with. So that's basically how we see our doctors [trainers] doing it and then we just follow that.* (SMS6.5, MBChB 6, female student, 33 years).

*… as part of our training also in Family Medicine, we have a week where we rotate [at] the GP’s practice. So, within that week you get the exposure as well on the continuity of care and you get to be exposed to the relationship between GPs and their patients.* (WSS5.7, MBChB 5, female student, 24 years old).

#### Challenges experienced by students

Students had experienced challenges in training on PHC in tertiary settings. They found the practical application of PHC learning lacking in other settings and the training in PHC was found to be implicit as there was no clear structure outlining it, especially among specialist disciplines. The meaning of this experience for students was clear to the researchers – they had expectations of hands-on practice in PHC to implement the theoretical knowledge they had been given by their trainers.

##### Training in tertiary health facilities

The training at tertiary institutions was mainly conducted by specialist trainers. Students were of the view that it deprived them the exposure to PHC training settings. This statement from students gave the researchers the sense that students had evaluated their training experience in the various training sites and could clearly state their (sites) differences for PHC training.

*… as medical students [we] are trained predominantly at tertiary institutions and those are on the opposite side of the spectrum to what primary health care is. So, we don't get much training in primary health care there… most of our exposure is to specialist care and tertiary care in the bigger academic hospitals as opposed to primary care.* (WTSM6.1, MBBCh 6, female student, 23 years).

*So, I feel if we had more time in a clinic, where we will … have better experience in terms of primary health care.* (SMS6.5, MBChB 6, female student, 33 years).

##### Practical application of PHC lacking

Students had experienced deficiency in the practical application of the theory they had been taught on PHC, such that they would find it difficult to apply the theory on their own if called upon to do so. This meant that students were looking for opportunities to implement what they had been taught theoretically on PHC. They had been frustrated by the lack of such opportunities.

*As for the primary health care, I think I can define some of the terms in the concept itself, but as for how I can apply it [PHC] and provide that kind of care, I would say "No."* (WTSM5.1, MBBCh 5, male student, 22 years). *… we are more theory-based and we lack practical skills.* (WTS5.4, MBBCh 5, male student, 23 years).

##### Training in PHC implicit

Students experienced training in PHC as implicit and left to the individual student to discover that a particular activity pertained to PHC, particularly in specialist disciplines. This conveyed the message to the researchers that training in PHC, as experienced by students, was not structured. It also meant that students were not being “sign posted” on PHC, guiding them properly on the implementation of PHC principles during the training.

*I think it [training in PHC] is more of an individual [student's responsibility] … we usually become… more of primary [health care] physicians when we are in Family Medicine. When we get to other blocks we become surgeons, we become specialists like they want us to be.* (SMS6.2, MBChB 6, male student, 25 years).

*I don't think it [training in PHC] is explicit. …[as] we focus mostly on the curative part,* (WTS5.4, MBBCh 5, male student, 23 years).

### Trainers

The trainers also viewed PHC as an approach to health care, shared their experiences of the contribution of generalists and specialists in PHC training, the first patient encounter with the healthcare system, a multidisciplinary approach inclusive of specialists and the provision of comprehensive care through health promotion and disease prevention. Like their students, the trainers also indicated the challenges they were encountering in training students in PHC.

#### Primary health care as an approach

The trainers conducted their training in PHC with the understanding that it was an approach to patient care, rather than a level of care. They regarded patient care as comprehensive, taking into consideration the social determinants of health which cut across every discipline. Regarding social determinants of health, the trainers indicated that they trained students to remember lifestyle modifications when managing patients. The student trainers had managed to impart this knowledge and practical exposure to their students as evidenced by students’ similar responses to PHC as an approach to health care delivery.

*We encourage our students, "when you write a discharge summary of this particular patient, always make sure on the discharge summary that you include lifestyle modifications. It's not just about writing paracetamol or augmentin or whatever it is. There has to be some concerted effort that you impart to the patient that he's going to adopt lifestyle modifications…”* (DWTT3, male trainer, Internal Medicine).

#### Training by generalists and specialists

##### Generalists

Family Medicine which is a generalist specialty, took the lead in the coordination of PHC training at the distributed training sites. A family physician indicated that they trained students to consult with any patient, regardless of their clinical condition, in PHC settings. In this way, family physicians regarded themselves as generalist medical practitioners, not confined to a specific area of practice.

*if a student has a patient who’s a pregnant woman who’s come for anti-natal care, we [as family physicians] are there, if the student says I’ve got little John here who’s 5 years old with severe malnutrition, we [as family physicians] are also there.* (AWST1, female trainer, Family Medicine).

##### Specialists

A surgeon also expressed surgeons’ involvement in PHC. The researchers inherited the understanding that specialists were aware of the importance of applying the PHC principle of prevention, for example screening patients before the condition is clinically detected.

*… they [surgeons] wrote a report saying seatbelts were needed … and eventually someone … implemented mandatory seatbelts and then they looked at the morality rate and the mortality rate went down. Now, that study was done by surgeons, it wasn’t done by a family physician or primary health – it was done by surgeons because [of] the burden of disease that they were seeing, … So, screening for cancer is an important role, screening for breast cancer in this country is a very important role because it’s such a common disease. So, educating around self-examination, those are things that we play a role in as surgeons and understand as surgeons because we know what the end role is. … So, I think that there’s lots that surgeons can have to offer in that primary health care preventative component …* (DWTT5, male trainer, General Surgery).

#### First encounter with the healthcare system

Student trainers indicated that they trained students on basic medical conditions commonly encountered and presenting firstly at PHC settings, using a multi-disciplinary approach. It became evident that student trainers viewed the “first encounter with patients” at PHC important to train their students on. This was intended to equip the students to recognize the basic conditions that can be dealt with at PHC.

*[At PHC], we teach them the basic mental illnesses that we come across, that are there in the communities on a daily basis, that’s what we do. And how they identify those basic mental illnesses, and also emphasise the importance of …a multidisciplinary approach in terms of mental health and illnesses in psychiatry.* (CSMT2, female trainer, Psychiatry).

#### Comprehensive care

Trainers conducted student training with the understanding that it was comprehensive (holistic) care, offering service in the best interest of the patient. Students had to adopt a comprehensive approach in dealing with patients at PHC, rationalizing on their referral decision in the context of a multidisciplinary team. Only complicated patient conditions warranted referral. The principle of comprehensive patient care was related to the principle of “ubuntu” which, literally translated means “I am because you are” in the African context. This interpretation translated into student training whereby they were taught to manage a patient holistically.

*So, in terms of the holistic approach, we train students to [respect the principle] of “Ubuntu” [comprehensive way of contexualising a patient’s condition] - we do it in a much broader way, … we try and practice it to give students [the] exposure …* (DWTT5, male trainer, General Surgery).

*So, for example, you see a patient who [needs] a lumber puncture, or you want to do thoracocentesis, … these are GP practices, you know… skills. Then they [students]‘ll tell you that “I’m going to wait for ehm… neurology to do a lumbar puncture.” I stopped them and said “No, the only time that you’re going for a neurologist [referral] is when you have a complicated case where you are not sure.” Now they do their own lumbar punctures.* (DWTT4, male trainer, Obstetrics and Gynaecology).

#### Coordination of care

The training was centered around the view that the generalist should take the lead in coordinating team-based care. To this end, students were trained on inter-professional collaboration where the primary health care practitioner encouraged team building for coordination of patient care. This principle conveyed the message of the importance of teamwork in clinical settings, discouraging treatment of patients in silos.

*Well, in my view [the doctor at primary health care] is the doctor that should lead the team [to offer coordinated health care services]. They should offer team-based care and leadership which isn't about "I run the team", but "how do I work with teams where I actually encourage leadership among the team members.”* (DWTT1, male trainer, Family Medicine).

*So, for example, we work with the psychologists, we work with the social workers on the floor. But for us, … we have what you call academic meetings, which students get involved in ….* (DWTT6, female trainer, Paediatrics).

#### Continuity of care

Training in this regard was achieved through longitudinal placement of student, whereby they would be in a training platform for an extended period of time. Student trainers also shared the perspective that PHC should be regarded as a continuum from district to tertiary health, that the care of a patient should be seamless from the PHC to tertiary institutions. The importance of a life-long relationship with a patient was hereby highlighted. The students were thus trained to develop interest in their patients demonstrated by follow-up enquiries, even if they refer patients to more specialized centers.

*… the training platform has to actually be based on the service platform. So you build up continuity [of care] through longitudinal attachment* (DWTT1, male trainer, Family Medicine).

… *in our 5th year of Family Medicine, we have decentralized learning where we take them to the health centres; part of it happens at district hospital level and part of it happens at a regional hospital, but most of it happens at the health centers.* (AWST1, female trainer, Family Medicine).

*So, I think that there’s lots that surgeons have to offer in that primary health care preventative component. Hepatitis B vaccine is so important to prevent hepatocellular carcinoma which becomes a surgical disease. We play a role in that continuum. That’s when I talk about systems, …* (DWTT5, male trainer, General Surgery).

#### Challenges experienced by student trainers

The trainers’ challenges in training students in PHC were mentioned as (1) those occasioned by training students at tertiary settings and (2) inadequacy of trainers at PHC settings. To the researchers this meant that the trainers were conscious of the inherent challenges in the process of training the students in PHC. The researchers further understood that some of these challenges could not be resolved by the trainers as they related to the training infrastructure.

##### Training in tertiary institutions

The challenge of training students at tertiary setting is that students are trained on patients devoid of primary health care symptoms, leading to theorization regarding the symptoms that patients presented with on arrival at PHC. Patient symptoms were already masked on arrival at tertiary settings, because of the clinical management already commenced at first encounter with patients and continued at secondary and tertiary settings.

*So, I think that is one of the challenges that we have. We get patients who are referred, who have already been treated …at lower levels of care. By the time they come here, the symptoms are already masked because… the patient has already been given treatment, and that creates a challenge.* (CSMT4, male trainer, Obstetrics and Gynaecology).

*… we are teaching them in an environment that is not a primary healthcare environment, we’re teaching them, for instance, here … they see patients at a regional level… We’re not teaching them about, uhm, if you were sitting at a primary health care clinic or in a community health clinic* (BKZT5, male trainer, General Surgery).

### Lack of trainers at distributed platforms

It was a challenge for one trainer to send students to distributed learning platforms where there would be no specialist in his field to ensure continuity of specialized training at those sights. This gave the sense that specialists would have appreciated to be capacitated to deploy their specialist colleagues to the distributed training sites to facilitate PHC.

*You see, in a sophisticated society in a first world environment these kids [medical students] would be looked after because wherever they go there would be someone there to hold their hands, but [in our case] where these kids are going there’s often no one there [in my field] to hold their hands.* (BKZT5, male trainer, General Surgery).

## Discussion

The study has outlined medical students’ and their trainers’ experiences in PHC training in accordance with the internationally known PHC attributes, their approach to PHC and the challenges they encountered in the training process.

### PHC as an approach

The understanding of PHC as an approach, rather than a level of healthcare delivery seemed to have influenced both the trainers and their students’ training experiences. For the students, they had experienced this approach when various disciplines (including specialists) empowered patients to actively participate in the management of their own conditions by educating them. Patient education is one of the focal points of PHC (31). Evidence indicates that empowering patients with self-efficacy through health education, defined as “one’s belief in one’s ability to succeed in specific situations,” improves health outcomes (32). The trainers also drew students’ attention to the importance of social determinants of health (SDH) which cut across all disciplines as an approach to healthcare delivery (33, 34). Trainers linked SDH, the benefits of which have been documented (35), to lifestyle modification practices for a comprehensive approach to patient care, instead of concentrating only on biomedical therapy. Therefore, the view of PHC as an approach to health care formed the requisite basis for student training on patient education and SDH (1, 36).

### Student trainers

Students had experienced the training on PHC as facilitated by both generalist and specialist trainers. They identified the discipline of Family Medicine as having played a major role in affording them the opportunity for exposure to PHC. There is evidence from other settings that the discipline of Family Medicine, as a generalist discipline, is well-place to train students in PHC (37, 38). However, the students were of the view that PHC training was not the sole responsibility of one department, but should be a shared responsibility among all departments, a view also supported by literature (39). Regarding the training on PHC, some students indicated that specialists tended to have a specialized rather than a comprehensive approach to patient care. This has led to the recommendation in other settings, that PHC be facilitated by generalists (40, 41). Family Medicine as a generalist specialty featured prominently in the training of students on PHC. However, specialists also indicated their contribution in this regard, as also backed by literature (42). The researchers in this study are of the view, in keeping with that of the students, that the training in PHC be a collaborative effort between generalists and specialists in each medical school.

### Point of first patient contact

Literature has indicated that PHC is the first patient contact with the health system (2, 41). Student training in this regard entailed their exposure to an undifferentiated patient which called for recognition of the clinical condition, stabilization of patients presenting with emergency conditions and referral of those who required management at more specialized centers (43). The student trainers addressed this attribute by training students on common clinical conditions frequently encountered at PHC settings using a multidisciplinary approach. A collaborative approach among disciplines with respect to patients presenting at the entry level of the health system has been found to be a useful clinical tool in addressing diverse common conditions at PHC (44). Again, students indicated that they had experienced the discipline of Family Medicine taking the lead in initiating to patients at the first point of entry to the health care system (45).

### Comprehensive care

Student training in comprehensive care entailed consideration of other factors that could influence the patient’s wellbeing. They indicated that they were trained not to be disease-focused, but to factor in the patient’s biopsychosocial context inclusive of the patient’s individual and contextual aspects, exemplified by the patient’s concerns and expectations about their conditions and community considerations, respectively. Globally, studies are beginning to show a move toward comprehensive patient care by all disciplines, including specialists (46, 47). Furthermore, students were also trained in health promotion and disease prevention which has been shown to ensure comprehensiveness in patient care (2, 48). The trainers tallied with their students in narrating their experiences in comprehensive care training. They emphasized patient-centered care based on rational clinical judgment, including patient referral to other disciplines (49). Students were encouraged to apply clinical reasoning, whereby every clinical decision taken is backed by evidence, which has been found to be the necessary skill toward comprehensive patient care (50, 51).

### Coordination of care

Coordinated care has been described as the integration of health care among providers “across levels of care and time” (52); care that is patient-centered, taking into consideration the patient’s needs and preferences (53), and sharing information among all the participants concerned with a patient’s care, aimed at achieving safer and more effective care (54). In this study, students narrated that various disciplines trained them on collaborative patient care, giving them insight on which patient’s condition should be attended to by which discipline, including receiving back patients who had initially been referred to other disciplines. The training was centered around the view that it is generalist practitioners at PHC facilities who should take the lead in coordinating team-based care (55). In this way, students were also introduced to inter-professional collaboration characterized by team building among health care practitioners (56, 57).

### Continuity of care

Students experienced training in continuity of patient care through their involvement in patient follow-up. Patient load and fewer trainers made it difficult for students to properly appreciate the implementation of this skill in the busy tertiary training facilities, but the attachment to a community general practitioner (GP) afforded them the opportunity. The patient congestion experienced in tertiary training institution has been reported elsewhere (58), and the positive role played by GPs in training students on continuity of care has also been documented (59). The student trainers indicated that they achieved training in continuity of care through.

longitudinal placement of students in decentralized training platforms. At one of the institutions, student placements took up to 6 months in their fifth year of training. In a study that compared the performance of students having community-based longitudinal placements to those in traditional placements in the medical school, it was found that the students in longitudinal placement were not disadvantaged by the placement—the performance of the two groups using the objective structured clinical examination was found to be comparable (60). Prior to this study, another study had obtained similar findings (61). Effective longitudinal placements have been found to integrate core disciplines while affording students the opportunity for immersion in the training sites (62).

### Challenges of students

Students narrated the challenges they faced regarding specialist participation in their training. Specialists encouraged the students to retrospectively reconstruct the initial patient presentation scenario, to imagine the signs and symptoms of the patient at the point of first presentation. However, that presented a challenge to students as the signs and symptoms of patients then presenting at tertiary level had already been masked by the management they had been receiving on their way to the tertiary platforms. Thus, in the students’ experience, training by specialists lacked the element of the undifferentiated patient with “red flags” (63), frequently encountered in PHC settings.

The other challenge pointed out by the students related to the implicitness of the training in PHC. They indicated that, during the training, it was left to the individual student to work out.

what would be applicable in PHC, particularly among specialist disciplines, where emphasis tended to be on specialized activities, and less so on PHC. Furthermore, although they had indicated that in their observation, PHC was applicable in every discipline they had rotated in, students found that very little effort was made by the trainers to highlight to them the scope that would be applicable in PHC. It was left unstructured, whereas in literature there is evidence of the effectiveness of a properly structured multi-disciplinary integrated PHC program (64).

### Challenges of trainers

Like their students, trainers also experienced challenges in student training on PHC. These were also occasioned by students’ training at tertiary settings and inadequacy of trainers at PHC settings. The trainers were also aware that at the tertiary training facilities, students encountered patients whose original symptoms and signs had already been masked. They tried improvisation by recreating the PHC patient presentation scenario. However, the specialist trainers acknowledged that students missed out on the hands-on experience at the point of patient entry to the health system (47). However, in spite of these tertiary training settings challenges, it must not be lost sight of that these settings are vital in exposing students to the endpoints of diseases as well as rare conditions (65). Furthermore, students had also expressed an opinion that PHC training should be the responsibility of each discipline. The view of this study researchers is that students and their trainers should maintain a continuous dialog on the signposting of information and skills applicable in PHC in each discipline that they rotate through.

The trainers, particularly specialists, also acknowledged that the number of specialist trainers in distributed training sites was inadequate. The specialists felt they needed to be physically involved in the decentralized training sites. Under these circumstances, literature has shown that outreach programs, whereby the university-based specialists visit students in the distributed platforms (66), as well as the enlisting of the services of community-based specialists, can support the requisite learning (67). This could also suggest that family medicine specialists (who adopt a generalist approach) could be entrusted by other specialists to attend to the specialist areas relevant for a medical practitioner who exits undergraduate training with a generalist overview of the practice of medicine (68). In a study conducted in South Africa, it has been demonstrated that Family Medicine can deliver a successful longitudinal integrated multidisciplinary clerkship among students (64).

### Learning contexts and settings

In the four medical schools the training platforms comprised central and distributed settings. It does appear that the distributed training platforms provide the required context for the training in PHC. For example, students indicated that at those settings, the training was mainly conducted by generalist medical practitioners with a PHC approach in patient care, including community GPs. The approach entailed mainly prevention of disease and promotion of health. The context was also conducive for the four pillars of Starfield (5), to be put into practice. Students and their trainers explicitly expressed their concern regarding training in PHC at tertiary institutions which they described as not ideal. While it is important to expose students.

to hospicentric medicine to familiarize them with the management of patients with advanced disease conditions (69), PHC training is best placed in distributed training settings which are mainly within communities (70).

### The experiences in PHC training vs. the understanding of PHC

In the previous publication on the “understanding” of the students and their trainers regarding PHC (authored by the same authors of this paper) (17), there was common understanding of PHC among them, in line with Starfield’s four pillars of PHC, namely comprehensiveness, first contact access, coordination and continuity of care (24).

There were also areas of convergence and divergence among the students as a group and their trainers as another group. Some among each group understood PHC as an approach to healthcare delivery, while others understood it as a level of care practiced only in PHC settings (community health facilities, including clinics and district hospitals). Strangely enough, in the current study, when exploring their experiences in PHC training, both groups had experienced PHC as an approach to healthcare delivery to patients. The latter is in keeping with the WHO definition of PHC in this regard (2). Therefore, there was a clear discrepancy in “experiences” and “understanding” of PHC in this regard which requires further exploratory studies.

Furthermore, in the previous publication there was no consensus in the understanding on whether specialists needed to be involved in training students in PHC, with some students understanding specialists as part of the training in PHC and others expressing uncertainty in.

this regard (17). The difference of opinions was also evident among the students’ trainers. However, in the current study students had experienced PHC training conducted by both generalists and specialists, although to a greater extent by generalists. With regard to students’ trainers, they expressed involvement in PHC training and the significance of doing so. It would seem that, regardless of the understanding of both students and their trainers, the practical experience what that both specialists and generalists were involved in student training in PHC. This involvement is backed by literature (71). It is the view of the authors of this current paper that there is need for a decisive development of a collaborative partnership between specialist and generalist trainers in PHC in the four South African medical schools.

### Study strengths and limitations

As far as the researchers of this study are aware, at the time when this study was conducted, there were none reporting on the experiences of students and their trainers in PHC in South African medical schools. However, the study was conducted in only four of the nine medical schools in the country at the time of the study. Although there might have been similarities between the selected medical schools and those which were left out, there might also have been significant differences between the two groups. This would therefore limit the transferability of this study findings to the other medical schools. Furthermore, students were not engaged on their assessment experiences in their training on PHC. This would have shed more light on their training experiences, as evidence indicates that assessment drives student learning (72).

## Conclusion

The study has shown that students and their trainers (generalists and specialists) had experienced UG student training in PHC in line with the internationally recognized principles on the subject. Compared to specialist disciplines, Family Medicine, as a generalist discipline, was found to be better placed in distributed training sites to train students on in PHC, although specialists also indicated their roles. The view by students and their trainers that PHC is an approach rather than a level of care enhanced its training across disciplines. Training students in PHC by specialists in tertiary settings presented a challenge to both students and their trainers. Students experienced training in PHC as implicit and unstructured. To optimize training in PHC, it is recommended that more time be dedicated to distributed PHC training sites and supportive specialist outreach training programs be ensured in the South African medical schools.

## Data availability statement

The datasets presented in this study can be found in online repositories. The names of the repository/repositories and accession number(s) can be found in the article/supplementary material.

## Ethics statement

The studies involving humans were approved by the Ethical Review Board of the University of KwaZulu-Natal in South Africa (Protocol reference number: HSS/2187/017D). The studies were conducted in accordance with the local legislation and institutional requirements. The participants provided their written informed consent to participate in this study. Written informed consent was obtained from the individual(s) for the publication of any potentially identifiable images or data included in this article.

## Author contributions

LM: Conceptualization, Data curation, Formal analysis, Investigation, Methodology, Resources, Validation, Visualization, Writing – original draft, Writing – review & editing. MM: Data curation, Formal analysis, Funding acquisition, Investigation, Methodology, Project administration, Resources, Supervision, Validation, Visualization, Writing – review & editing.

## References

[1] World Health Organisation. Declaration of Alma-Ata. International conference on primary Health care, Alma-Ata, USSR, 6–12 September 1978. World Health Organisation, Geneva, (1978). Available at: https://www.who.int/teams/social-determinants-of-health/declaration-of-alma-ata (Accessed 11 October 2019).

[2] World Health Organization and United Nations Children's Fund (UNICEF). A vision for primary health care in the 21st century: Towards universal health coverage and the sustainable development goals. World Health Organization. (2018). Available at: https://www.who.int/docs/default-source/primary-health/vision.pdf (Accessed 6 March 2023).

[3] Generalist Task Force Education Strategy. Report of the Generalism and generalist task force education strategy, innovations, and development unit. Royal College of Physicians and Surgeons of Canada, Ottawa, Ontario, (2013). Available at: http://www.royalcollege.ca/rcsite/educational-initiatives/generalism-medical-education-e (Accessed 11 October 2017).

[4] Abbas SM Alam AY Malik R. Primary health care: what is it and what is it not? Views of teaching faculty at an undergraduate medical college in Pakistan. East Mediterr Health J. (2012) 18:261–4. doi: 10.26719/2012.18.3.261 22574481

[5] Starfield B. Primary care: Balancing Health needs, services and technology,2nd Edn. New York and Oxford: Oxford University Press, (1998):8–9.

[6] Coovadia H Jewkes R Barron P Sanders D McIntyre D. The health and health system of South Africa: historical roots of current public health challenges. Lancet. (2009) 374:817–34. doi: 10.1016/S0140-6736(09)60951-X, PMID: 19709728

[7] PHASA. The implementation of PHC re-engineering in South Africa. Professional hunters Association of South Africa (PHASA), (2015). Available at: https://phasa.org.za/2011/11/15/the-implementation-of-phc-re-engineering-in-south-africa/ (Accessed 15 May 2020).

[8] The World Bank. Accountability in Public Services in South Africa. (2011). Available at: https://documents.worldbank.org/en/publication/documents-reports/documentdetail/707121468164063748/south-africa-accountability-in-public-services-in-south-africa (Accessed 10 September 2020).

[9] Fox JA. Social accountability: what does the evidence really say? World Dev. (2015) 72:346–61. doi: 10.1016/j.worlddev.2015.03.011, PMID: 38618829

[10] Besigye I Mash R Essuman A Flinkenflogel M. Undergraduate family medicine and primary care training in sub-Saharan Africa: reflections of the PRIMAFAMED network. Afr J Prim Health Care Fam Med. (2017) 9:e1–5. doi: 10.4102/phcfm.v9i1.1351, PMID: 28155289 PMC5291078

[11] Macinko J Montenegro H Nebot Adell C Etienne C. Renewing primary health care in the Americas. Rev Panam Salud Publica. (2007) 21:73–84. doi: 10.1590/S1020-49892007000200003 17565795

[12] Bhate TD Loh LC. Building a generation of physician advocates: the case for including mandatory training in advocacy in Canadian medical school curricula. Acad Med. (2015) 90:1602–6. doi: 10.1097/ACM.0000000000000841, PMID: 26200573

[13] Jimenez G Matchar D Koh GCH Tyagi S van der Kleij RMJJ Chavannes NH. Revisiting the four core functions (4Cs) of primary care: operational definitions and complexities. Prim Health Care Res Dev. (2021) 22:e68–9. doi: 10.1017/S1463423621000669, PMID: 34753531 PMC8581591

[14] Moshabela M Moodley N Campbell C Gaede B Flack P Diab P. Social accountability in the transformation of medical education to meet the needs of health care systems and local communities in South Africa. University of KwaZulu-Natal, Durban (2015). Available at: https://copsam.com/wp-content/uploads/2015/12/Sa-and-transformation-in-Medical-education.pdf (Accessed 6 April 2019).

[15] Van Rooyen M. Using fourth-year medical students’ reflections to propose strategies for primary care physicians, who host students in their practices, to optimise learning opportunities. S Afr Fam Pract. (2012) 54:513–7. doi: 10.1080/20786204.2012.10874285

[16] Draper CE Louw G. Primary health care in the South African context—medical students’ perspectives: open forum. SA Fam Pract. (2007) 49:6–11. doi: 10.10520/EJC80037

[17] Mabuza LH Moshabela M. What do medical students and their clinical preceptors understand by primary health care in South Africa? A qualitative study. BMC Med Educ. (2023) 23:785. doi: 10.1186/s12909-023-04751-x, PMID: 37864172 PMC10589924

[18] Bahari SF. Qualitative versus quantitative research strategies: contrasting epistemological and ontological assumptions. J Teknol. (2010) 52:17–28. doi: 10.11113/sh.v52n1.256

[19] Terre Blanche M Durrheim K Painter D. Research in practice: Applied methods for the social sciences. 2nd ed. Cape Town: University of Cape Town Press (2006).

[20] Creswell JW. Qualitative Inquiry and Research Design: Choosing among the five approaches. Thousand Oaks, CA: SAGE Publications, Inc; (2013). pp. 77–83.

[21] Azungah T. Qualitative research: deductive and inductive approaches to data analysis. Qual Res J. (2018) 18:383–400. doi: 10.1108/QRJ-D-18-00035, PMID: 38831594

[22] Shenton AK. Strategies for ensuring trustworthiness in qualitative research projects. Educ Inf. (2004) 22:63–75. doi: 10.3233/EFI-2004-22201

[23] Pearse N. An illustration of deductive analysis in qualitative research. Rhodes University, Grahamstown, South Africa: In *The 18th European Conference on Research Methodology for Business and Management Studies ECRM, Hosted by the Wits Business School, Johannesburg, South Africa*. (2019). Available at: https://eprints.lincoln.ac.uk/id/eprint/36421/1/ECRM19-Proceedings-Download.pdf#page=279 (Accessed 29 July 2023).

[24] Foo C Surendran S Jimenez G Ansah JP Matchar DB Koh GCH. Primary care networks and Starfield's 4Cs: a case for enhanced chronic disease management. Int J Environ Res Public Health. (2021) 18:2926. doi: 10.3390/ijerph18062926, PMID: 33809295 PMC8001119

[25] Vygotsky LS. Mind in society: The development of higher psychological processes. Cambridge, MA: Harvard University Press (1978).

[26] Drew C. Situated learning theory (Lave & Wegner) – pros & cons (2020). Available at: https://helpfulprofessor.com/situated-learning-theory/ (Accessed 26 April 2020).

[27] Besar PH. Situated learning theory: the key to effective classroom teaching? IJECS. (2018) 1:49–60.

[28] Anfara VAJ Brown KM Mangione TL. Qualitative analysis on stage: making the research process more public. Educ Res. (2002) 31:28–38. doi: 10.3102/0013189X031007028, PMID: 38737938

[29] Younas A Fàbregues S Durante A Escalante E Inayat S Ali P. Proposing the “MIRACLE” narrative framework for providing thick description in qualitative research. Int J Qual Methods. (2023) 22:160940692211471. doi: 10.1177/16094069221147162

[30] Creswell JW. Educational research: Planning, conducting and evaluating quantitative and qualitative research, upper Saddle River. NJ: Merrill Prentice Hall (2012).

[31] van Weel C Kidd MR. Why strengthening primary health care is essential to achieving universal health coverage. CMAJ. (2018) 190:E463–6. doi: 10.1503/cmaj.170784, PMID: 29661815 PMC5903888

[32] Paterick TE Patel N Tajik AJ Chandrasekaran K. Improving health outcomes through patient education and partnerships with patients. Proc. (2017) 30:112–3. doi: 10.1080/08998280.2017.11929552, PMID: 28152110 PMC5242136

[33] Gard LA Cooper AJ Youmans Q Didwania A Persell SD Jean-Jacques M. Identifying and addressing social determinants of health in outpatient practice: results of a program-wide survey of internal and family medicine residents. BMC Med Educ. (2020) 20:18. doi: 10.1186/s12909-020-1931-1 31948434 PMC6966817

[34] Moore DE Jr Green JS Gallis HA. Achieving desired results and improved outcomes: integrating planning and assessment throughout learning activities. J Contin Educ Heal Prof. (2009) 29:1–15. doi: 10.1002/chp.20001, PMID: 19288562

[35] Braveman P Gottlieb L. The social determinants of health: it's time to consider the causes of the causes. Public Health Rep. (2014) 129 Suppl 2:19–31. doi: 10.1177/00333549141291S206, PMID: 24385661 PMC3863696

[36] World Health Organization. Primary health care: Closing the gap between public health and primary care through integration. Geneva, Switzerland: World Health Organization (2018).

[37] Bradner M Crossman SH Vanderbilt AA Gary J Munson P. Career advising in family medicine: a theoretical framework for structuring the medical student/faculty advisor interview. Med Educ Online. (2013) 18:1. doi: 10.3402/meo.v18i0.21173, PMID: 23948497 PMC3756303

[38] Kardonsky K Evans DV Erickson J Kost A. Impact of a targeted rural and underserved track on medical student match into family medicine and other needed workforce specialties. Fam Med. (2021) 53:111–7. doi: 10.22454/FamMed.2021.351484, PMID: 33566345

[39] Irlam J Keikelame MJ Vivian L. Integrating the primary health care approach into a medical curriculum: a programme logic model. Afr J Health Prof Educ. (2010) 1:8–10.

[40] Rosenthal J Stephenson A. General practice: the future training environment: a report on undergraduate primary care education in London. Br J Gen Pract. (2010) 60:144. doi: 10.3399/bjgp10X483391, PMID: 20132726 PMC2814286

[41] Silverston P. Consultation skills teaching in primary care. Educ Prim Care. (2013) 24:206–18. doi: 10.1080/14739879.2013.11494174 23676878

[42] Girotto LC Enns SC de Oliveira MS Mayer FB Perotta B Santos IS. Preceptors’ perception of their role as educators and professionals in a health system. BMC Med Educ. (2019) 19:1–8. doi: 10.1186/s12909-019-1642-7 31196069 PMC6567907

[43] Brekke M Carelli F Zarbailov N Javashvili G Wilm S Timonen M. Undergraduate medical education in general practice/family medicine throughout Europe: a descriptive study. BMC Med Educ. (2013) 13:157. doi: 10.1186/1472-6920-13-157, PMID: 24289459 PMC4220558

[44] Flinkenflögel LM Kyamanywa P Cubaka VK Cotton P. The next generation of Rwandan physicians with a primary health care mindset. Afr J Prm Health Care Fam Med. (2015) 7:2. doi: 10.4102/phcfm.v7i1.885 PMC4564837 26245625

[45] Mudiyanse RM. Need to teach family medicine concepts even before establishing such practice in a country. Asia Pac Fam Med. (2014) 13:1. doi: 10.1186/1447-056X-13-1, PMID: 24397851 PMC3904475

[46] Kendrick T. 30th George swift lecture: generalism in undergraduate medical education: what's next? Br J Gen Pract. (2012) 62:323–5. doi: 10.3399/bjgp12X649296, PMID: 22687227 PMC3361114

[47] Donisi V Gajofatto A Mazzi MA Gobbin F Busch IM Ghellere A. A bio-PsychoSocial co-created intervention for young adults with multiple sclerosis (ESPRIMO): rationale and study protocol for a feasibility study. Front Psychol. (2021) 12:215. doi: 10.3389/fpsyg.2021.598726 PMC7940381 33708157

[48] Gibson G. What can the treatment of Parkinson’s disease learn from dementia care; applying a bio-psycho-social approach to Parkinson’s disease. Int J Older People Nursing. (2017) 12:1–8. doi: 10.1111/opn.12159 28685944

[49] Nicholls G Blythe A Pearson D. Making an educational case for a national primary care curriculum for medical students: lessons from one UK medical school. Educ. Prim Care. (2012) 23:313–6. doi: 10.1080/14739879.2012.11494130, PMID: 23182093

[50] Gay S Bartlett M McKinley R. Teaching clinical reasoning to medical students. Clin Teach. (2013) 10:308–12. doi: 10.1111/tct.12043, PMID: 24015736

[51] Kinsinger FS. Beneficence and the professional's moral imperative. J Chiropr Human. (2009) 16:44–6. doi: 10.1016/j.echu.2010.02.006, PMID: 22693466 PMC3342811

[52] Rao M Pilot E. The missing link--the role of primary care in global health. Glob Health Action. (2014) 7:23693. doi: 10.3402/gha.v7.23693 24560266 PMC3926992

[53] Feng W Feng X Shen P Wang Z Wang B Shen J. Influence of the integrated delivery system on the medical serviceability of primary hospitals. J Healthc Eng. (2021) 2021:9950163. doi: 10.1155/2021/9950163, PMID: 34394901 PMC8356014

[54] Primary Health Care Performance Initiative (PHCPI). Improvement strategies model: high quality primary health care: coordination. Version 1.0 (2019). Available at: https://www.improvingphc.org/sites/default/files/Coordination%20May%202019.pdf (Accessed 9 November 2023).

[55] Hashim MJ. Principles of family medicine and general practice–defining the five core values of the specialty. J Prim Health Care. (2016) 8:283–7. doi: 10.1071/HC16006, PMID: 29530151

[56] Care Coordination Agency for Healthcare Research and Quality, Rockville, MD. (2018). Available at: https://www.ahrq.gov/ncepcr/care/coordination.html (Accessed 9 November 2023).

[57] Bentley M Freeman T Baum F Javanparast S. Interprofessional teamwork in comprehensive primary healthcare services: findings from a mixed methods study. J Interprof Care. (2018) 32:274–83. doi: 10.1080/13561820.2017.1401986, PMID: 29182411

[58] Strasser R Couper I Wynn-Jones J Rourke J Chater AB Reid S. Education for rural practice in rural practice. Educ Prim Care. (2016) 27:10–4. doi: 10.1080/14739879.2015.1128684, PMID: 26862793

[59] Xiao YT. Exposure of Chinese undergraduates to general practice teaching. Br J Gen Pract. (2017) 67:252–3. doi: 10.3399/bjgp17X691013, PMID: 28546395 PMC5442930

[60] Hari R Harris M Frey P Streit S. Broadening the clinical spectrum for medical students towards primary care: a pre-post analysis of the effect of the implementation of a longitudinal clerkship in general practice. BMC Med Educ. (2018) 18:34. doi: 10.1186/s12909-018-1152-z, PMID: 29540163 PMC5853096

[61] Frattarelli LC Kamemoto LE. Obstetrics and gynecology medical student outcomes: longitudinal multispecialty clerkship versus traditional block rotations. Am J Obstet Gynecol. (2004) 191:1800–4. doi: 10.1016/j.ajog.2004.07.066, PMID: 15547568

[62] Thistlethwaite JE Bartle E Chong AAL Dick M-L King D Mahoney S. A review of longitudinal community and hospital placements in medical education: BEME guide no. 26. Med Teach X. (2013) 35:e1340–64. doi: 10.3109/0142159X.2013.806981, PMID: 23848374

[63] Ramanayake RP Basnayake BM. Evaluation of red flags minimizes missing serious diseases in primary care. J Family Med Prim Care. (2018) 7:315–8. doi: 10.4103/jfmpc.jfmpc_510_15, PMID: 30090770 PMC6060920

[64] de Villiers M Conradie H van Schalkwyk S. Teaching medical students in a new rural longitudinal clerkship: opportunities and constraints. Ann Glob Health. (2018) 84:58–65. doi: 10.29024/aogh.17, PMID: 30873776 PMC6748171

[65] Campbell J Hobbs FDR Irish B Nicholson S Pringle M Reeve J. UK academic general practice and primary care visible? Viable? Invaluable BMJ. (2015) 351:h4164. doi: 10.1136/bmj.h4164 26231511

[66] Nyangari B Couper ID Sondzaba NO. Exposure to primary healthcare for medical students: experiences of final-year medical students. SA Fam Pract. (2010) 52:467–70. doi: 10.1080/20786204.2010.10874027

[67] Battye KM White C Cronin S Bond N Mitchell C. Developing solutions to the provision of primary care services in rural and remote Queensland. *8th National Rural Health Conference*. (2022) Available at: https://ruralhealth.org.au/8thNRHC/Papers/battye_cronin.pdf (Accessed 5 March 2022).

[68] HPCSA: Health Professions Act 56 of 1974. Regulations relating to the registration of students, undergraduate curricula and professional examinations in medicine” published under government notice R139 in government gazette 31886 of 19 February (2009). Available at: https://www.gov.za/sites/default/files/gcis_document/201409/31886139.pdf (Accessed 10 June 2023).

[69] Rockey NG Ramos GP Romanski S Bierle D Bartlett M Halland M. Patient participation in medical student teaching: a survey of hospital patients. BMC Med Educ. (2020) 20:142. doi: 10.1186/s12909-020-02052-1, PMID: 32381082 PMC7206775

[70] Gizaw Z Astale T Kassie GM. What improves access to primary healthcare services in rural communities? A systematic review. BMC Prim Care. (2022) 23:313. doi: 10.1186/s12875-022-01919-0, PMID: 36474184 PMC9724256

[71] Leinster S. Training medical practitioners: which comes first, the generalist or the specialist? J R Soc Med. (2014) 107:99–102. doi: 10.1177/0141076813519438, PMID: 24526462 PMC3938126

[72] Burch V Reid S. “Fit for purpose?” the appropriate education of health professionals in South Africa. Afr Med J. (2011) 101:25–6. doi: 10.7196/SAMJ.4695 21626976

