# Supplementary material for: What are the experiences of medical students and their trainers regarding undergraduate training in primary health care at four South African medical schools? A qualitative study
Source: Front Med (Lausanne). 2024 Jun 18;11:1337140. doi: 10.3389/fmed.2024.1337140 (PMC11217328; doi:10.3389/fmed.2024.1337140)
Supplement: Supplementary file 1 [file Appendix_A_clinical_preceptors.docx]

**Appendix A**

**Students’ clinical preceptors in the four medical schools**

|  | **HODs** | **Discipline** | **Code** |
| --- | --- | --- | --- |
|  | **Institution A** |  |  |
|  |  | Family Medicine | AWST1 |
|  |  | Internal Medicine | AWST2 |
|  |  | Obstetrics & Gynaecology | AWST3 |
|  |  | General Surgery | AWST4 |
|  |  | Paediatrics & Child Health | AWST5 |
|  |  | Dermatology | AWST6 |
|  |  |  |  |
|  | **Institution B** |  |  |
|  |  | Family Medicine | BKZT1 |
|  |  | Psychiatry | BKZT2 |
|  |  | Internal Medicine | BKZT3 |
|  |  | Obstetrics & Gynaecology | BKZT4 |
|  |  | General Surgery | BKZT5 |
|  |  | Paediatrics & Child Health | BKZT6 |
|  |  | Orthopaedics | BKZT7 |
|  |  |  |  |
|  | **Institution C** |  |  |
|  |  | Family Medicine | CSMT1 |
|  |  | Psychiatry | CSMT2 |
|  |  | Internal Medicine | CSMT3 |
|  |  | Obstetrics & Gynaecology | CSMT4 |
|  |  | General Surgery | CSMT5 |
|  |  | Paediatrics & Child Health | CSMT6 |
|  |  | Orthopaedics | CSMT7 |
|  |  |  |  |
|  | **Institution D** |  |  |
|  |  | Family Medicine | DWTT1 |
|  |  | Psychiatry | DWTT2 |
|  |  | Internal Medicine | DWTT3 |
|  |  | Obstetrics & Gynaecology | DWTT4 |
|  |  | General Surgery | DWTT5 |
|  |  | Paediatrics & Child Health | DWTT6 |
|  |  | Orthopaedics | DWTT7 |
