# Supplementary material for: What are the experiences of medical students and their trainers regarding undergraduate training in primary health care at four South African medical schools? A qualitative study
Source: Front Med (Lausanne). 2024 Jun 18;11:1337140. doi: 10.3389/fmed.2024.1337140 (PMC11217328; doi:10.3389/fmed.2024.1337140)
Supplement: Supplementary file 2 [file Appendix_B_students.docx]

**Appendix B**

**Medical students from the four medical schools**

| **MBChB 4** | | | | **MBChB 5** | | | | **MBChB 6** | | | |
| --- | --- | --- | --- | --- | --- | --- | --- | --- | --- | --- | --- |
| **Student Number** | **Code** | **Sex** | **Age**  **(years)** | **Student**  **Number** | **Code** | **Sex** | **Age**  **(years)** | **Student**  **Number** | **Code** | **Sex** | **Age**  **(years)** |
|  | WSS4.1 | F | 20 | 36. | WSS5.1 | M | 24 | 69. | WSS6.1 | F | 23 |
|  | WSS4.2 | M | 21 | 37. | WSS5.2 | F | 23 | 70. | WSS6.2 | F | 28 |
|  | WSS4.3 | M | 24 | 38. | WSS5.3 | F | 23 | 71. | WSS6.3 | M | 24 |
|  | WSS4.4 | F | 22 | 39. | WSS5.4 | F | 24 | 72. | WSS6.4 | M | 26 |
|  | WSS4.5 | F | 20 | 40. | WSS5.5 | M | 22 | 73. | WSS6.5 | M | 25 |
|  | WSS4.6 | F | 22 | 41. | WSS5.6 | M | 27 | 74. | WSS6.6 | F | 24 |
|  | WSS4.7 | M | 25 | 42. | WSS5.7 | F | 24 | 75. | WSS6.7 | F | 26 |
|  | WSS4.8 | M | 23 | 43. | WSS5.8 | M | 23 | 76. | WSS6.8 | F | 27 |
|  | WSS4.9 | F | 23 | 44. | WSSM5.1 | M | 23 | 77. | WSSM6.1 | M | 29 |
|  | WSSM4.1 | M | 25 | 45. | WSSM5.2 | F | 22 | 78. | KZS6.1 | M | 23 |
|  | WSSM4.2 | F | 27 | 46. | KZS5.1 | M | 23 | 79. | KZS6.2 | M | 24 |
|  | WSSM4.3 | M | 33 | 47. | KZS5.2 | F | 24 | 80. | KZS6.3 | F | 26 |
|  | KZS4.1 | M | 21 | 48. | KZS5.3 | F | 23 | 81. | KZS6.4 | M | 24 |
|  | KZS4.2 | F | 21 | 49. | KZS5.4 | F | 23 | 82. | KZS6.5 | F | 25 |
|  | KZS4.3 | M | 22 | 50. | KZS5.5 | F | 22 | 83. | KZS6.6 | M | 24 |
|  | KZS4.4 | M | 21 | 51. | KZS5.6 | M | 24 | 84. | KZS6.7 | F | 24 |
|  | KZS4.5 | F | 26 | 52. | KZS5.7 | F | 25 | 85. | KZS6.8 | M | 25 |
|  | KZS4.6 | M | 21 | 53. | KZSM5.1 | F | 23 | 86. | KZS6.9 | F | 24 |
|  | KZS4.7 | M | 22 | 54. | KZSM5.2 | M | 22 | 87. | KZS6.10 | M | 26 |
|  | KZS4.8 | F | 23 | 55. | SMS5.1 | M | 36 | 88. | KZSM6.1 | F | 23 |
|  | KZSM4.1 | M | 23 | 56. | SMS5.2 | M | 30 | 89. | KZSM6.2 | F | 24 |
|  | KZSM4.2 | M | 21 | 57. | SMS5.3 | M | 41 | 90. | SMS6.1 | M | 24 |
|  | SMS4.1 | F | 22 | 58. | SMS5.4 | F | 23 | 91. | SMS6.2 | M | 25 |
|  | SMS4.2 | F | 21 | 59. | SMS5.5 | M | 29 | 92. | SMS6.3 | M | 28 |
|  | SMS4.3 | M | 22 | 60. | SMSM5.1 | F | 21 | 93. | SMS6.4 | M | 25 |
|  | SMS4.4 | M | 21 | 61. | SMSM5.2 | M | 23 | 94. | SMS6.5 | F | 33 |
|  | SMS4.5 | M | 20 | 62. | WTS5.1 | M | 22 | 95. | SMSM6.1 | M | 24 |
|  | SMSM4.1 | M | 21 | 63. | WTS5.2 | M | 22 | 96. | SMSM6.2 | M | 29 |
|  | SMSM4.2 | M | 21 | 64. | WTS5.3 | F | 23 | 97. | SMSM6.3 | M | 23 |
|  | WTS4.1 | M | 23 | 65. | WTS5.4 | M | 23 | 98. | WTS6.1 | M | 23 |
|  | WTS4.2 | M | 25 | 66. | WTS5.5 | M | 23 | 99. | WTS6.2 | M | 22 |
|  | WTS4.3 | M | 24 | 67. | WTSM5.1 | M | 21 | 100. | WTS6.3 | M | 25 |
|  | WTS4.4 | F | 21 | 68. | WTSM5.2 | M | 22 | 101. | WTS6.4 | M | 23 |
|  | WTS4.5 | M | 22 |  |  |  |  | 102. | WTSM6.1 | F | 23 |
|  | WTSM4.1 | F | 20 |  |  |  |  |  |  |  |  |
