# Supplementary material for: What are the experiences of medical students and their trainers regarding undergraduate training in primary health care at four South African medical schools? A qualitative study
Source: Front Med (Lausanne). 2024 Jun 18;11:1337140. doi: 10.3389/fmed.2024.1337140 (PMC11217328; doi:10.3389/fmed.2024.1337140)
Supplement: Supplementary file 3 [file Appendix_C_Dataset.docx]

**Appendix C**

**Information for participant validation**

Dear Participant

This is the combined information obtained through the interviews conducted by the researchers in the four medical schools in South Africa to which you participated.

Kindly make comments and send through to the Principal Investigator at [honeymanyosi@gmail.com](mailto:honeymanyosi@gmail.com)

Study title:

**What are the experiences of medical students and their trainers regarding undergraduate training in primary health care at four South African medical schools? A qualitative study**

**Themes and sub-themes:**

| 1. **Students** 2. PHC – an approach to patient care 3. Training by generalists and specialists    1. Generalists    2. Specialists 4. First contact 5. Comprehensive care 6. Coordination of care 7. Continuity of care 8. Challenges experienced    1. Training in tertiary health facilities    2. Practical application of PHC lacking    3. Training in PHC implicit | 1. **Trainers** 2. PHC – an approach to patient care 3. Training by generalists and specialists   2.1. Generalists  2.2. Specialists   1. First contact 2. Comprehensive care 3. Coordination of care 4. Continuity of care6. 5. Challenges experienced   7.1. Training in tertiary institutions  7.2. Lack of trainers at distributed platforms |
| --- | --- |

1. **Students**

Students described their experiences in the training in PHC by identifying their trainers,

PHC as the first contact of patient care, comprehensive care, coordination of care and continuity of care. They also narrated the challenges they had experienced during the training.

1. **Experience of PHC as an approach**

Students indicated that they had experienced PHC as an approach rather than a level of patient care, and as an approach, they had observed that some specialists (not only the generalists) had practiced PHC in their various disciplines by educating patients on disease prevention, which indicated to the students that PHC cuts across all disciplines and is not confined to the patient entry levels which were the clinics and other community based health facilities.

*Yeah, with the primary health care being a care, not a level, … most specialists do practice it, I have experienced [it] in the surgery block where they educated the patient about the, the prevention of a condition. Also, in psychiatry also I had experienced it,…* (SMSM4.1, MBChB 4, female student, 21 years).

1. **Generalist and specialist trainers in PHC**

In their experiences of being trained by generalists and specialists, students compared these experiences.

- 1. **Generalists**

According to the students, generalist practice trainers offered training more than specialists in PHC. However, they also felt that PHC should be trained by all disciplines and not only confined to generalist trainers.

*…it's essentially the general practitioner who has a greater caliber of skills to be able to address primary health care as opposed to the surgeon.* (WTSM5.2, MBChB 5, male student, 22 years).

The discipline of Family Medicine was identified as particularly giving students the opportunity of exposure to PHC.

*I think it’s mostly Family Medicine, honestly speaking, our Family Medicine rotation is the one that focusses a lot on it [training in PHC]. In fourth year, we are in clinics, in community health care centres and then in fifth year we are in a district hospital but in a rural area.* (KZS5.1, MBChB 5, male students, 23 years).

However, students expressed further opinion on the responsible disciplines in training students on PHC.

*It [teaching primary health care] is supposed to be a sort of shared responsibility among the departments.* (SMS5.4, MBChB 5, female student, 23 years).

- 1. **Specialists**

Some students had experienced being trained by specialists on PHC, through reconstruction of the state of the patient on arrival at PHC before referral to specialised disciplines. However, some students also indicated that they had not experienced training in PHC from specialist disciplines because of the specialised setting.

*In my case when we went there, they did [train us on PHC]. They often asked us "what would lead to this? And what did you expect to see before the patient came in?". And then there are certain signs that started presenting before, which could give you a clue towards the diagnosis.* (WTS4.3, MBBCh 4, male student, 24 years).

*So, they often asked us to, to not only think about what the patient is presenting with now, but also what they might have presented with before, so that ... in case we saw a patient at a primary health care setting, that we would uhm... have enough knowledge to pick up a case and either treat it or refer it upwards.* (WTS4.4, MBBCh 4, female student, 21 years).

*because obviously if you're doing an Obs and Gyne [Obstetrics & Gynaecology] block for example, and you’re in a Gyne [Gynaecological] ward, … you don't really want to explore the other problems.* (KZS4.5, MBChB 4, female student, 26 years).

1. **First contact**

Students pointed out that they were given guidance on the approach to patients seen at PHC settings at entry points into the healthcare system. They were taught to apply basic principles of recognition of a condition, stabilization of emergencies and appropriate referral determined by limitations.

*[At primary health care] you have to be able to recognize these conditions and refer where possible. Yeah, and up to so far, yeah, they emphasize on that, yeah, we are being taught that when it gets here, there's nothing you can do … make sure that they’re breathing, give them oxygen, give them fluids and then refer, …* (SMS5.5, MBChB 5 female student, 29 years old).

1. **Comprehensive care**

Training in comprehensive care entailed consideration of other factors that could influence the patient’s wellbeing: the social determinants of health. This enabled the students to address the patient’s condition comprehensively, not to lose focus on all the aspects that could affect their wellbeing.

*Yeah, we do get trained on comprehensive patient care. It's about treating the patient, everything that has to do with the patient. So, it's not only treating the disease – the biological part of it. So, you consider the social background of the patient, you consider the psychological issues that they might have also. So, if the patient, say you are treating an HIV or TB patient, … maybe they live in a house where they can infect other people, where they can infect children. So, you need to also deal with that, the social background.* (WTS5.3, MBBCh 5, female student, 25 years).

*So, for social determinants of health, we get taught to include a social history, and a social history will include things like … family history. But we also need to make sure that we look at the patient holistically in terms of occupation, finances, access to clean water, electricity, and all of those things that can affect the patient's health in the long term.* (SMS6.3, MBCHB 6, male students, 28 years).

1. **Coordination of care**

Various disciplines trained students on collaborative patient care, giving them insight on which patient’s condition should be attended to by which discipline, including receiving back patients who had initially been referred to other disciplines.

*…they do teach us … to be able to identify different conditions, … knowing who is responsible or who can help on which particular condition and where is that person. So, you need to know and understand what a dietitian does, you need to know and understand what all these specialists to do. We know if I have a patient who is a diabetic … they need to change their lifestyles, I have to refer them to someone who will give them proper exercises and [diet] schedules …* (WTS5.5, MBBCh 5, male student, 23 years).

*We are also taught that you don't only send them, you also have to check on their progress while they are still there [where you referred them to] because they'll have to come back to you again after they are done.* (KZSM5.2, MBChB 5, male student, 23 years).

1. **Continuity of care**

Continuity of patient care entailed arrangement of patient follow-up. GP attachment gave them the opportunity for training in continuity of care.

*Well of course we are trained to … make follow-up, so we ask them [patients] to come back after [a] certain period of time based on the condition ... they are having problems with. So that's basically how we see our doctors [trainers] doing it and then we just follow that.* (SMS6.5, MBChB 6, female student, 33 years).

*… as part of our training also in Family Medicine, we have a week where we rotate [at] the GP’s practice. So, within that week you get the exposure as well on the continuity of care and you get to be exposed to the relationship between GPs and their patients.* (WSS5.7, MBChB 5, female student, 24 years old).

1. **Challenges experienced by students**

Students had experienced challenges in training on PHC in tertiary settings. They found the practical application of PHC learning lacking in other settings and the training in PHC was found to be implicit as there was no clear structure outlining it, especially among specialist disciplines.

- 1. **Training in tertiary health facilities**

The training at tertiary institutions was mainly conducted by specialist trainers. Students were of the view that it deprived them the exposure to PHC training settings:

*... as medical students [we] are trained predominantly at tertiary institutions and those are on the opposite side of the spectrum to what primary health care is. So, we don't get much training in primary health care there... most of our exposure is to specialist care and tertiary care in the bigger academic hospitals as opposed to primary care.* (WTSM6.1, MBBCh 6, female student, 23 years).

*So, I feel if we had more time in a clinic, where we will … have better experience in terms of primary health care.* (SMS6.5, MBChB 6, female student, 33 years).

- 1. **Practical application of PHC lacking**

Students had experienced deficiency in the practical application of the theory they had been taught on PHC, such that they would find it difficult to apply the theory on their own if called upon to do so:

*As for the primary health care, I think I can define some of the terms in the concept itself, but as for how I can apply it [PHC] and provide that kind of care, I would say "No".* (WTSM5.1, MBBCh 5, male student, 22 years). *… we are more theory-based and we lack practical skills.* (WTS5.4, MBBCh 5, male student, 23 years).

- 1. **Training in PHC implicit**

Students experienced training in PHC as implicit and left to the individual student to discover that a particular activity pertained to PHC, particularly in specialist disciplines:

*I think it [training in PHC] is more of an individual [student's responsibility]... we usually become... more of primary [health care] physicians when we are in Family Medicine. When we get to other blocks we become surgeons, we become specialists like they want us to be.* (SMS6.2, MBChB 6, male student, 25 years).

*I don't think it [training in PHC] is explicit. …[as] we focus mostly on the curative part,* (WTS5.4, MBBCh 5, male student, 23 years).

1. **Trainers**

The trainers also viewed PHC as an approach to health care, the contribution of generalists and specialists in PHC training, the first patient encounter with the healthcare system, a multidisciplinary approach inclusive of specialists and the provision of comprehensive care through health promotion and disease prevention. Like their students, the trainers also indicated the challenges they were encountering in training students in PHC.

1. **Primary health care as an approach**

The trainers conducted their training in PHC with the understanding that it was an approach to patient care, rather than a level of care. They regarded patient care as comprehensive, taking into consideration the social determinants of health which cut across every discipline. Regarding social determinants of health, the trainers indicated that they trained students to remember lifestyle modifications when managing patients:

*We encourage our students, "when you write a discharge summary of this particular patient, always make sure on the discharge summary that you include lifestyle modifications. It's not just about writing paracetamol or augmentin or whatever it is. There has to be some concerted effort that you impart to the patient that he's going to adopt lifestyle modifications…”* (DWTT3, male trainer, Internal Medicine).

1. **Training by generalists and specialists**

**2.1. Generalists**

Family Medicine which is a generalist specialty, took the lead in the coordination of PHC training at the distributed training sites. A family physician indicated that they trained students to consult with any patient, regardless of their clinical condition, in PHC settings.

*if a student has a patient who’s a pregnant woman who’s come for anti-natal care, we* *[as family physicians] are there, if the student says I’ve got little John here who’s 5 years old with severe malnutrition, we [as family physicians] are also there.* (AWST1, female trainer, Family Medicine).

**2.2. Specialists**

A surgeon also expressed surgeons’ involvement in PHC

*… they [surgeons] wrote a report saying seatbelts were needed … and eventually someone … implemented mandatory seatbelts and then they looked at the morality rate and the mortality rate went down. Now, that study was done by surgeons, it wasn’t done by a family physician or primary health – it was done by surgeons because [of] the burden of disease that they were seeing, … So, screening for cancer is an important role, screening for breast cancer in this country is a very important role because it’s such a common disease. So, educating around self-examination, those are things that we play a role in as surgeons and understand as surgeons because we know what the end role is. … So, I think that there’s lots that surgeons can have to oﬀer in that primary health care preventative component …* (DWTT5 , male trainer, General Surgery).

1. **First encounter with the healthcare system**

Student trainers indicated that they trained students on basic medical conditions commonly encountered and presenting firstly at PHC settings, using a multi-disciplinary approach:

*[At PHC], we teach them the basic mental illnesses that we come across, that are there in the communities on a daily basis, that’s what we do. And how they identify those basic mental illnesses, and also emphasise the importance of …a multidisciplinary approach in terms of mental health and illnesses in psychiatry.* (CSMT2, female trainer, Psychiatry).

1. **Comprehensive care**

Trainers conducted student training with the understanding that it was comprehensive (holistic) care, offering service in the best interest of the patient. Students had to adopt a comprehensive approach in dealing with patients at PHC, rationalizing on their referral decision in the context of a multidisciplinary team. Only complicated patient conditions warranted referral:

*So, in terms of the holistic approach, we train student to [respect the principle] of “Ubuntu” [comprehensive way of contexualising a patient’s condition] - we do it in a much broader way, … we try and practice it to give students [the] exposure …* (DWTT5, male trainer, General Surgery).

*So, for example, you see a patient who [needs] a lumber puncture, or you want to do thoracocentesis, … these are GP practices, you know… skills. Then they [students]’ll tell you that “I’m going to wait for ehm… neurology to do a lumbar puncture.” I stopped them and said “No, the only time that you’re going for a neurologist [referral] is when you have a complicated case where you are not sure.” Now they do their own lumbar punctures.* (DWTT4, male trainer, Obstetrics and Gynaecology).

1. **Coordination of care**

The training was centred around the view that the generalist should take the lead in coordinating team-based care. To this end, students were trained on inter-professional collaboration where the primary health care practitioner encouraged team building for coordination of patient care.

*Well, in my view [the doctor at primary health care] is the doctor that should lead the team [to offer coordinated health care services]. They should offer team-based care and leadership which isn't about "I run the team", but "how do I work with teams where I actually encourage leadership among the team members.”* (DWTT1, male trainer, Family Medicine).

*So, for example, we work with the psychologists, we work with the social workers on the floor. But for us, … we have what you call academic meetings, which students get involved in ….* (DWTT6, female trainer, Paediatrics).

1. **Continuity of care**

Training in this regard was achieved through longitudinal placement of student, whereby they would be in a training platform for an extended period of time. Student trainers also shared the perspective that PHC should be regarded as a continuum from district to tertiary health, that the care of a patient should be seamless from the PHC to tertiary institutions.

*… the training platform has to actually be based on the service platform. So you build up continuity [of care] through longitudinal attachment* (DWTT1, male trainer, Family Medicine).

… *in our 5th year of Family Medicine, we have decentralized learning where we take them to the health centres; part of it happens at district hospital level and part of it happens at a regional hospital, but most of it happens at the health centers.* (AWST1, female trainer, Family Medicine).

*So, I think that there’s lots that surgeons have to oﬀer in that primary health care preventative component. Hepatitis B vaccine is so important to prevent hepatocellular carcinoma which becomes a surgical disease. We play a role in that continuum. That’s when I talk about systems, …* (DWTT5, male trainer, General Surgery).

1. **Challenges experienced by student trainers**

The trainers’ challenges in training students in PHC were mentioned as (1) those occasioned by training students at tertiary settings, (2) inadequacy of trainers at PHC settings, and (3) inappropriate PHC teaching approaches.

- 1. **Training in tertiary institutions**

The challenges mentioned were (1) training students on patients devoid of primary health care symptoms, leading to theorization regarding the symptoms that patients presented with on arrival at PHC. Patient symptoms were already masked on arrival at tertiary setting, because of the clinical management already commenced at first encounter with patients and continued as secondary and tertiary settings.

*So, I think that is one of the challenges that we have. We get patients who are referred, who have already been treated …at lower levels of care. By the time they come here, the symptoms are already masked because… the patient has already been given treatment, and that creates a challenge.* (CSMT4, male trainer, Obstetrics and Gynaecology).

*… we are teaching them in an environment that is not a primary healthcare environment, we’re teaching them, for instance, here … they see patients at a regional level… We’re not teaching them about, uhm, if you were sitting at a primary health care clinic or in a community health clinic* (BKZT5, male trainer, General Surgery).

- 1. **Lack of trainers at distributed platforms**

It was a challenge for one trainer to send students to distributed learning platforms where there would be no specialist in his field to ensure continuity of specialised training at those sights.

*You see, in a sophisticated society in a first world environment these kids [medical students] would be looked after because wherever they go there would be someone there to hold their hands, but [in our case] where these kids are going there’s often no one there [in my field] to hold their hands.* (BKZT5, male trainer, General Surgery).
